# Mind the Gap: Upgrading Genomes with Pacific Biosciences RS Long-Read Sequencing Technology

**DOI:** 10.1371/journal.pone.0047768

**Published:** 2012-11-21

**Authors:** Adam C. English, Stephen Richards, Yi Han, Min Wang, Vanesa Vee, Jiaxin Qu, Xiang Qin, Donna M. Muzny, Jeffrey G. Reid, Kim C. Worley, Richard A. Gibbs

**Affiliations:** Department of Molecular and Human Genetics, Human Genome Sequencing Center, Baylor College of Medicine, Houston, Texas, United States of America; Auburn University, United States of America

## Abstract

Many genomes have been sequenced to high-quality draft status using Sanger capillary electrophoresis and/or newer short-read sequence data and whole genome assembly techniques. However, even the best draft genomes contain gaps and other imperfections due to limitations in the input data and the techniques used to build draft assemblies. Sequencing biases, repetitive genomic features, genomic polymorphism, and other complicating factors all come together to make some regions difficult or impossible to assemble. Traditionally, draft genomes were upgraded to “phase 3 finished” status using time-consuming and expensive Sanger-based manual finishing processes. For more facile assembly and automated finishing of draft genomes, we present here an automated approach to finishing using long-reads from the Pacific Biosciences RS (PacBio) platform. Our algorithm and associated software tool, PBJelly, (publicly available at https://sourceforge.net/projects/pb-jelly/) automates the finishing process using long sequence reads in a reference-guided assembly process. PBJelly also provides “lift-over” co-ordinate tables to easily port existing annotations to the upgraded assembly. Using PBJelly and long PacBio reads, we upgraded the draft genome sequences of a simulated *Drosophila melanogaster*, the version 2 draft *Drosophila pseudoobscura*, an assembly of the Assemblathon 2.0 budgerigar dataset, and a preliminary assembly of the Sooty mangabey. With 24× mapped coverage of PacBio long-reads, we addressed 99% of gaps and were able to close 69% and improve 12% of all gaps in *D. pseudoobscura*. With 4× mapped coverage of PacBio long-reads we saw reads address 63% of gaps in our budgerigar assembly, of which 32% were closed and 63% improved. With 6.8× mapped coverage of mangabey PacBio long-reads we addressed 97% of gaps and closed 66% of addressed gaps and improved 19%. The accuracy of gap closure was validated by comparison to Sanger sequencing on gaps from the original *D. pseudoobscura* draft assembly and shown to be dependent on initial reference quality.

## Introduction

Genome finishing has become a lost art due to the expense of oligonucleotide directed Sanger sequencing relative to the low cost-per-base of second generation sequencing technologies. The first generation of large eukaryotic model organism genome sequencing projects, such as *Drosophila melanogaster* [1], *Caenorhabditis elegans* [2], *Arabidopsis thaliana* [3], human [4] , and mouse [5], all relied on a mapped bacterial artificial chromosome (BAC) approach. In the BAC approach, individual mapped BACs were shotgun sequenced, assembled, and manually finished before being pieced together creating the final, finished reference genome. Because of the prohibitive cost and labor required for BAC library creation, arraying, mapping, and preparation of subclone libraries from tens of thousands of BACs, these techniques fell out of favor. They were replaced by significantly less expensive and time-consuming whole genome assembly methods. Initially, assembly methods used relatively long (500–800 bp) shotgun Sanger reads with Overlap-Layout-Consensus assemblers [6]–[11]. Due to financial considerations, Sanger whole genome assemblies often used as few reads as possible, saving millions of dollars, but producing lower quality genomes using as little as 6× genome coverage, falling significantly short of the 10–15× required for high quality draft assemblies.

Second-generation genome assemblies have been based on shorter read, massively parallel sequencing technologies and De Bruijn graph assembly techniques [12]–[15] Depending on the dataset quality, polymorphism, and repetitiveness of the target genome, both approaches generate draft genomes with contig N50 sizes ranging from 5 kb–200 kb. Assemblies with short contig N50 statistics suffer from having many gene models (the foundation of most biological research) with gaps, missing exons, genes split between scaffolds, or missing entirely. As an anecdotal example, consider the rhesus macaque – an important biomedical model organism with a large international research community. The current draft of the rhesus macaque genome contains sequence gaps in up to 20% of its gene models. Most other eukaryotic genomes larger than yeast are currently assembled only to draft genome quality and have similar problems of varying degree (Table 1). The scale of the unfinished genome problem will be compounded by new initiatives to sequence 10,000 vertebrate genomes (http://www.genome10k.org/), 5,000 arthropod genomes (http://arthropodgenomes.org/wiki/i5K) and 1,000 additional plant and animal genomes (http://ldl.genomics.org.cn/page/pa-research.jsp.)

**Table 1:** Gap numbers and size distributions for representative high quality draft assemblies of highly studied species.

Organism	Common Name	% Bases in Gaps	Mean Gap Size	Median Gap Size
Apis mellifera	Honey Bee	8.40%	1892	54
Equus caballus	Horse	1.80%	1010	282
Gallus gallus	Chicken	1.30%	1267	302
Glycine max	Soy Bean	1.80%	1208	176
Macaca mulatta	Rhesus macaque	7.20%	1513	374
Monodelphis domestica	Opossum	2.50%	1699	386
Ornithorhynchus anatinus	Platypus	7.70%	585	249
Pan troglodytes	Chimpanzee	6.70%	1799	539
Pongo abelii	Orangutan	6.40%	852	428
Rattus norvegicus	Brown Rat	8.90%	1590	57
Sus scrofa	Wild hog	10.30%	1218	100
Strongylocentrotus purpuratus	Purple Sea Urchin	12.80%	844	50

Historically, several approaches have been used to upgrade draft genome sequences in a cost effective and automated manner. Early on, cosmids and BACs were assembled from forward only reads, and subclones pointing into gaps were then selected for reverse sequencing to reduce primer design costs and enable easier automation [16]. As capillary electrophoresis techniques evolved, Sanger reads as long as 1 kb were used to aid gap closure in an approach similar to ours (LI-COR Biosciences Lincoln NE). With the advent of massively-parallel short read technologies, paired reads of multiple insert sizes have been used to “reach” into gaps from unique contig sequences – for example Atlas-GapFill and the SOAP-denovo gap filler both use this approach.

It is important to note the distinctions between genome upgrading & finishing, and simple re-assembly. While better assembly algorithms may allow incremental improvements in re-assembling a given data set, the resulting contigs and scaffolds will have no relationship to previous assembly versions, thus losing existing annotations. In contrast, genome upgrading fills gaps and upgrades low quality regions, preserving most of the assembled sequence and annotations. To fully exploit the improved assembly, annotations must be re-evaluated in regions with improved sequence and newly closed gaps, but this is unnecessary in the majority of the assembly.

The Pacific Biosciences RS (PacBio) is the first sequencing technology to offer very long read lengths (average read lengths are 2–3 kb and >7 kb reads are not uncommon) without GC-bias or systematic errors, though it suffers the shortcomings of modest throughput and low accuracy (∼15% error rate skewed toward insertions). Despite these issues, extremely long and unbiased reads are uniquely suited for upgrading genomes. We developed a software tool (PBJelly) that uses PacBio reads to close gaps and preserve annotations. We applied PBJelly to four draft genome assemblies (one from simulated data), creating improved versions of these genomes and “lift-over” tables for annotation preservation.

## Results

### Design and Implementation of PBJelly

PBJelly is an automated pipeline for gap filling and genome improvement that aligns long sequence reads to draft assembles in order to close or improve captured gaps. PBJelly is currently applicable to PacBio RS reads, but it can be generalized to apply to any long-reads. PBJelly was designed and implemented with the goals of making genome upgrades automatic, fast, accurate, and reproducible. PBJelly trusts the input draft genome to be accurate and functions to improve what is already known about the genome, modifying the existing draft as little as possible by focusing on gaps and regions with missing and/or low-quality data. Furthermore, PBJelly verbosely logs all improvements to the draft genome, which enables identification and rejection of questionable gap fills, and production of an annotation co-ordinates lift-over table. Finally, PBJelly can be run on any cluster (we use Moab/Torque/PBS) parallelizing the gap filling process for rapid turn around.

**Figure 1:**
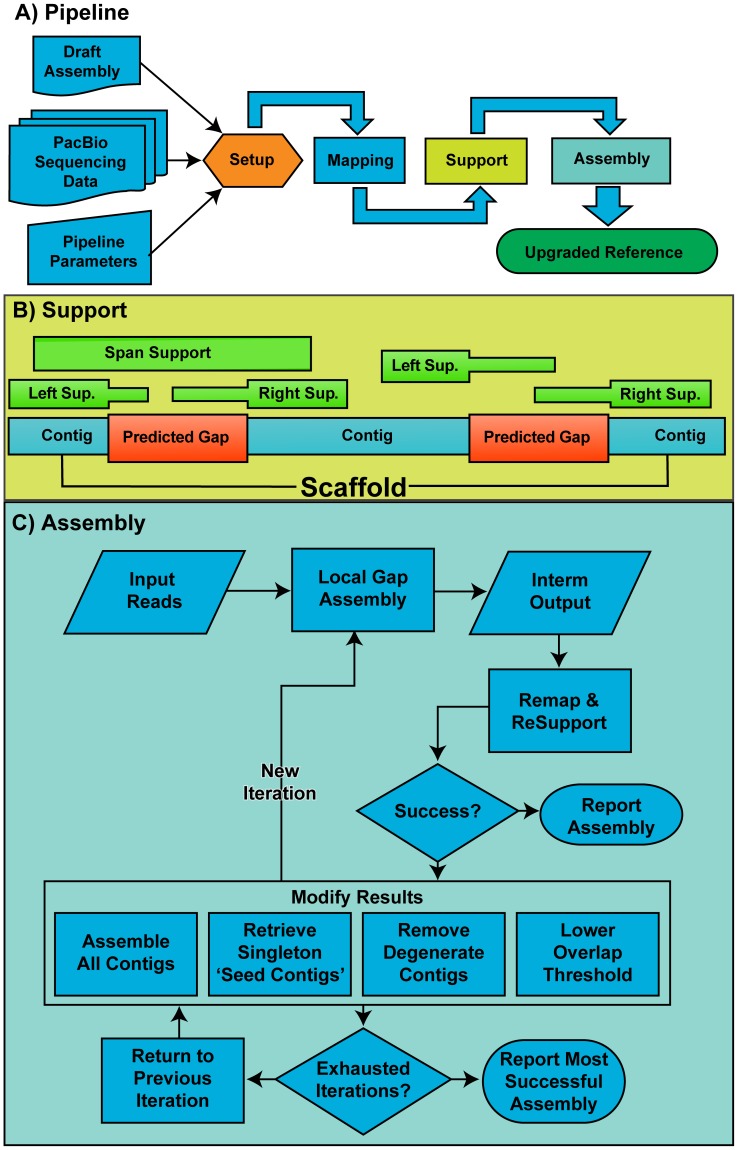
A schematic of PBJelly's workflow and decision-making. (A) A flow chart of PBJelly's steps. (B) A schematic describing two hypothetical gaps supported by reads and the classifications used during the Support step. (C) A detailed flow chart for local assembly of PacBio reads in a gap region used during the assembly step.

Figure 1a diagrams the PBJelly workflow. PBJelly begins with a “Setup” process that imports scaffold sequences from a reference genome and automatically identifies gaps. Any stretch of 25 or more N's within a scaffold defines a gap. Low-quality regions consist of consecutive N's shorter than 25 bp in length.

After setup, PBJelly maps the long-reads to the reference using BLASR (Basic Local Alignment and Serial Refinement) [17], which was specifically designed with the PacBio data error model in mind, though BLASR can align, reads in fasta format from any sequencing technology. The BLASR alignment information is parsed by the support procedure and serves dual purposes, identifying sequence adapters and reads that address gaps.

A side effect of sequencing double-stranded DNA templates [18] with a high error-rate is that occasionally the hairpin adapters on the ends of the template are not properly identified and removed. Retaining an adapter creates a single read with a particular mapping geometry composed of two subreads that overlap on opposite strands. We have seen this occur in approximately 1% of PacBio reads. PBJelly can identify these reads by looking at the multi-mapping information. A single read with multiple overlapping alignments to the reference that have similar start and/or end positions on opposite strands is indicative of a missed adapter. PBJelly uses these coordinates to split a read into the two separate subreads flanking the adapter.

The second function of the support procedure is to determine which reads address gaps by comparing aligned and un-aligned base positions within each read. In the simplest case, a single read will span a gap with an alignment to both the right and left flanking sequences (Figure 1b). PBJelly considers the alignment score of the entire gap-spanning read, though longer gaps with many Ns may lower the alignment score below the acceptable threshold since the alignment penalizes regions with missing data. To identify all possible gap supporting reads, PBJelly parses multi-mapping information where read alignment to each side of the gap is scored as a separate alignment. Given a 15% error rate, PBJelly requires ∼200 bp of sequence aligned to the contig ends flanking a gap, and at least 25 bp of un-aligned sequence mapping into the gap for a sequence read to be used for gap assembly.

In cases where the gap is too large for a single read to span PBJelly identifies reads reaching into the gap by aligning to and extending the gap's flanking contig sequences. A read must satisfy two criteria to be considered a candidate for gap support in this way: (1) the read alignment to the flanking sequence must match to within 25 bp of the start of the gap and (2) the read must have a minimum of 25 un-aligned bases that reach into the gap. This flank-extension approach allows gaps that don't have a single, spanning read to still be closed if the reads extending the flanking contigs are long enough to overlap and assemble into a single contig (Figure 1b). If the gap is too large for flank-extension reads to assemble across the gap, PBJelly incorporates the flank-extension reads and reduces the size of the gap.

After the gap-supporting sequence reads are identified, PBJelly assembles the reads for each gap to generate a high quality gap-filling consensus sequence. The assembly process is illustrated in Figure 1c. Each local gap assembly is fed the raw sequence of reads supporting a particular gap as well as information about the predicted gap size. Additionally, 1 kb of reference sequence from the flanking contigs are treated as reads and consolidated with the gap-supporting input reads. All this is collected by PBJelly and assembled using the Pacific Biosciences *de novo* assembly engine, ALLORA (Pacific Biosciences Menlo Park, CA). This overlap-layout-consensus (OLC) assembly engine is based on the AMOS open source suite [19].

The contigs produced that are composed of at least one of the flanking contig sub-sequences are then identified as seed contigs. By treating these sub-sequences as input reads for the OLC Assembly we provide a guide for the gap supporting reads' overlap and layout. PBJelly then re-maps and scores the seed contigs and decides if the new local assembly accurately fills the gap. PBJelly currently has two main criteria for measuring the accuracy of a local assembly. The first metric checks if the assembly supports the gap in the same fashion as was discovered in the support step. For example, if reads were identified to span the gap, an accurate assembly would also span the gap. The second metric compares the amount of sequence placed into the gap with the predicted size of the gap. For example, if a contig is built that closes a gap but would place several times more sequence into the gap than the gap's predicted size, PBJelly tries to find another assembly of the input reads that would fill the gap with a contig similar in length to the predicted gap size.

When an accurate assembly is found, PBJelly reports the gap-filling sequence. If a putative gap-filling seed contig produced in one iteration does not meet the accuracy criteria for gap filling, PBJelly chooses among a set of operations that modify the batch of sequences produced before passing them to the next iteration of the OLC Assembly. The operations include (1) removing degenerate contigs (i.e. ‘excess’ contigs that do not have an identifiable overlap with at least one of the original flanking contigs), (2) retrieving and adding singleton contigs that were not reported by the assembly and were generated from the reference gap-flanking sequence (usually due to other reads having a stronger overlap/layout connection to one ‘seed contig’ than the other), (3) using relaxed overlap parameters and re-assembling the input sequences. If all of these operations are exhausted and an appropriate gap assembly has yet to be found, PBJelly returns to a previous assembly iteration and modifies its produced contigs with the next available operation. This iterative approach, along with a proper assessment technique of how well a particular assembly fills a gap, employs a backtracking algorithm to find an optimal solution (Figure 1c). Finally, consensus gap filling sequences are simply spliced into the gap position in the draft assembly, replacing all N's if the gap is closed and leaving the appropriate number of N's if the gap is only reduced.

### Applying PBJelly to Draft Genomes

**Figure 2:**
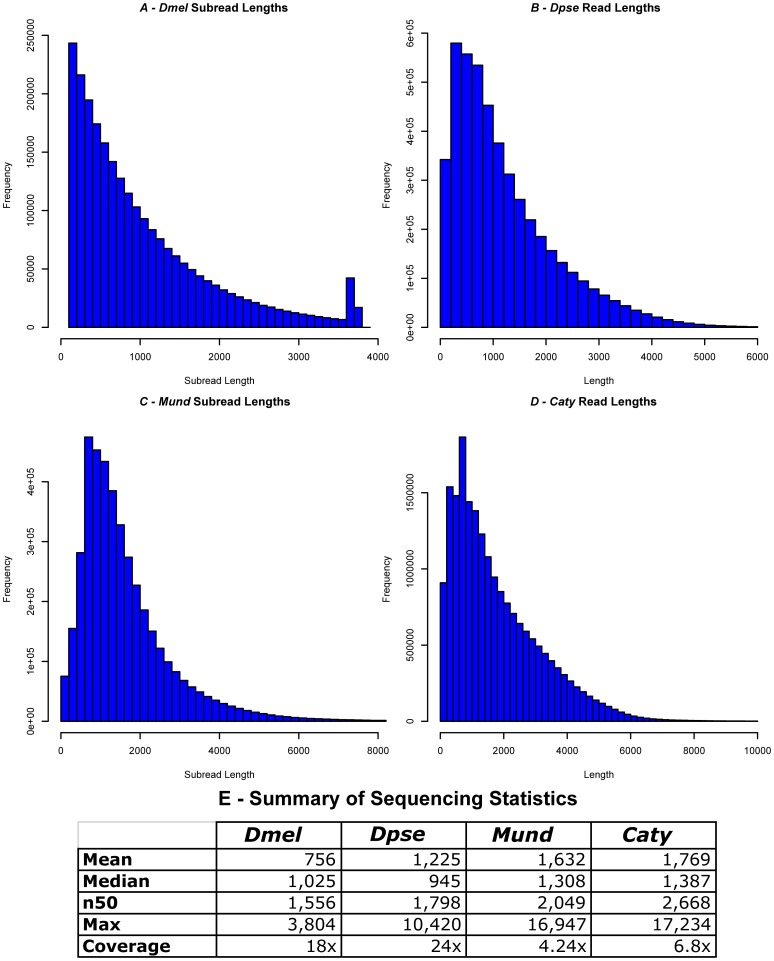
Description of sequencing data sets used. Histograms of read lengths in (A) Dmel, (B) Dpse, (C) Mund, (D) Caty. Panel (E) contains detailed metrics of each dataset.

Four datasets were used to assess the utility of PBJelly and PacBio long-reads in automatically finishing existing draft genomes. The first dataset was 18× mapped-coverage PacBio data simulated from the finished *Drosophila melanogaster* (*Dmel*) at an error-rate similar to that produced by the PacBio RS (average read accuracy of 85% and an error profile of 2% mismatches, 4% deletions, and 9% insertions.) We then ‘degraded’ the quality of the *Dmel* reference by randomly inserting gaps of various lengths. The second dataset was 24× mapped-coverage of *Drosophila pseudoobscura* (*Dpse*) sequence generated on the PacBio instrument for the *Dpse* 2.0 assembly. The *Dpse* DNA used for library construction was the same extraction used for the original draft genome sequencing [20]. We participated in the Assemblathon 2.0, (http://assemblathon.org/) which provided PacBio and other sequence data for the *Melopsittacus undulates (Mund)* genome project. We generated and submitted a PacBio free assembly for the competition, which is also used here for improvement. Finally, we also worked to improve a preliminary assembly of the sooty mangabey *Cercocebus atys (Caty)* by incorporating 6.8× mapped-coverage of PacBio data. All data sets are described in Figure 2. The *Dpse* and *Caty* data sets were filtered for minimum quality of 0.75 and minimum read length of 50 bp using PacBio SMRTAnalysis software. The *Dmel* and *Mund* data sets were provided only as subreads (i.e. no SMRTBell adapters).

**Figure 3:**
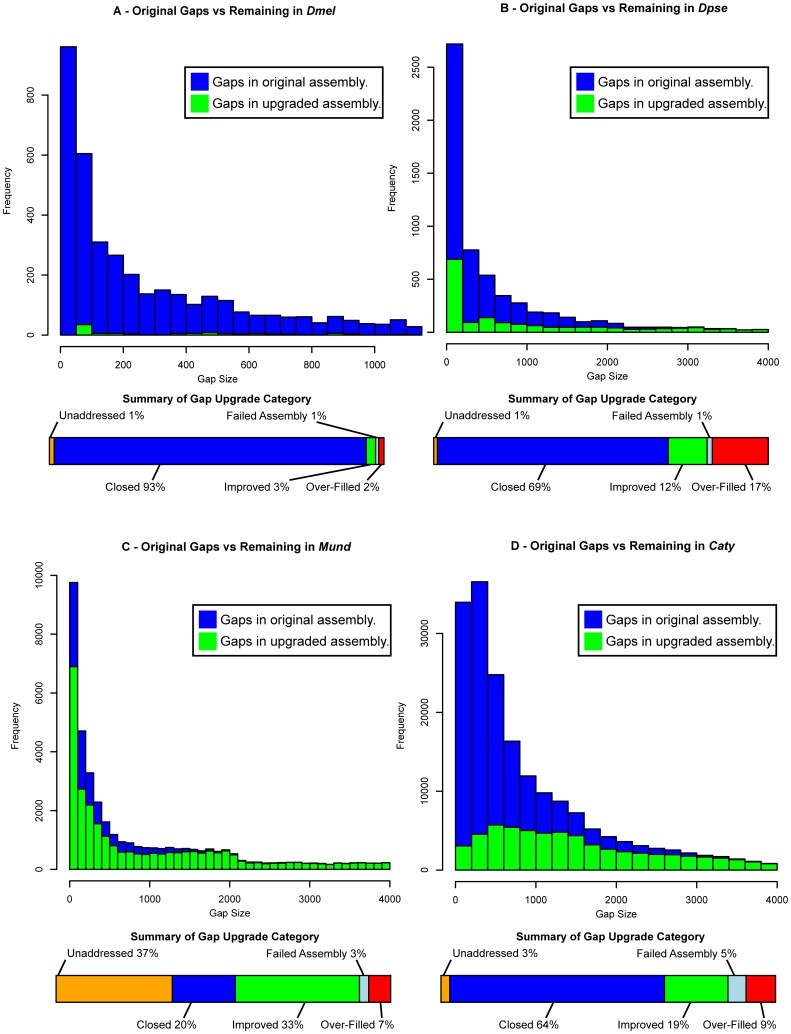
Gap filling Improvements and categories produced by PBJelly. Histograms showing gap-size distribution in the original and upgraded (A) D .mel, (B) Dpse, (C) Mund, and (D) Caty references as well as a summary of the upgrade categories for gaps.

Our PBJelly software (described below) implements read alignment, filtering, gap sequence assembly, and gap filling to improve existing assemblies. A gap is considered closed when it's neighboring contigs are connected by constructed sequence. A gap is improved by extending the neighboring contigs into the gap, although the entire gap sequence remains unresolved. Figure 3 describes the gaps before and after applying our method. The *Dmel* simulated draft had 4,651 gaps covering 3.19 Mb randomly and artificially inserted into it. Performing gap filling with 18× mapped coverage of simulated PacBio reads, we closed 93.25% of the gaps and improved another 2.8%. The *Dpse* 2.0 assembly had 6,026 gaps covering 6.67 Mb. After gap filling, 69% of the gaps were closed and 11.58% were improved, the total gap size was reduced by 54.1%, the contig N50 increased from 53 kb to 224 kb, and the total contig size increased by 3 Mb (1.96% of the genome). Our *Mund* assembly had 49,376 gaps spanning 155 Mb. After gap filling with 4.24× mapped coverage, 20.16% of the gaps were closed, 39.72% of the gaps were improved, the total gap size was reduced by 20.3 Mb (13%), the total contig size increased by 20 Mb, and the contig N50 increased from 134 kb to 233 kb. For our preliminary *Caty* assembly, with 6.8× mapped-coverage, we closed 64.18% of gaps and improved 18.95%. This increased the contig N50 from 35 kb to 128 kb. Table 2 lists the complete statistics for the assemblies before and after PBJelly's improvements. For clarity in our reports, only one interation of PBJelly was applied to the datasets.

**Table 2:** Gap Fill Statistics for PBJelly.

Dmel	Original	Upgraded	Improvement
Gap Count	4,651	311	15.0×
Gap n50	1,815 bp	3,504 bp	1.9×
Total Gap Size	3.19 Mb	541.3 Kb	5.9×
Contig n50	64,006 bp	723,621 bp	11.3×
Total Contig Size	133.6 Mb	136.3 Mb	1.0×

Dpse	Original	Upgraded	Improvement
Gap Count	6,026	1,852	3.3×
Gap n50	3,703 bp	15,919 bp	4.3×
Total Gap Size	6.67 Mb	3.61 Mb	1.8×
Contig n50	53,051 bp	224,350 bp	4.2×
Total Contig Size	146 Mb	149 Mb	1.0×

Mund	Original	Upgraded	Improvement
Gap Count	49,376	39,204	1.3×
Gap n50	10,546 bp	10,852 bp	1.0×
Total Gap Size	154.9 Mb	134.6 Mb	1.2×
Contig n50	134,382 bp	233,269 bp	1.7×
Total Contig Size	1.17 Gb	1.19 Gb	1.0×

Caty	Original	Upgraded	Improvement
Gap Count	186,841	66,211	2.8×
Gap n50	1,998 bp	2,052 bp	1.0×
Total Gap Size	197.5 Mb	79.3 Mb	2.5×
Contig n50	34,925 bp	128,379 bp	3.7×
Total Contig Size	2.64 Gb	2.76 Gb	1.0×

To facilitate the transfer of annotations from previous assemblies to the PBJelly gap filled assemblies, lift-over tables with co-ordinate shifts and descriptions of the changes made to the original assembly are generated alongside the upgraded reference.

### Validation of gap-closing sequences

**Figure 4:**
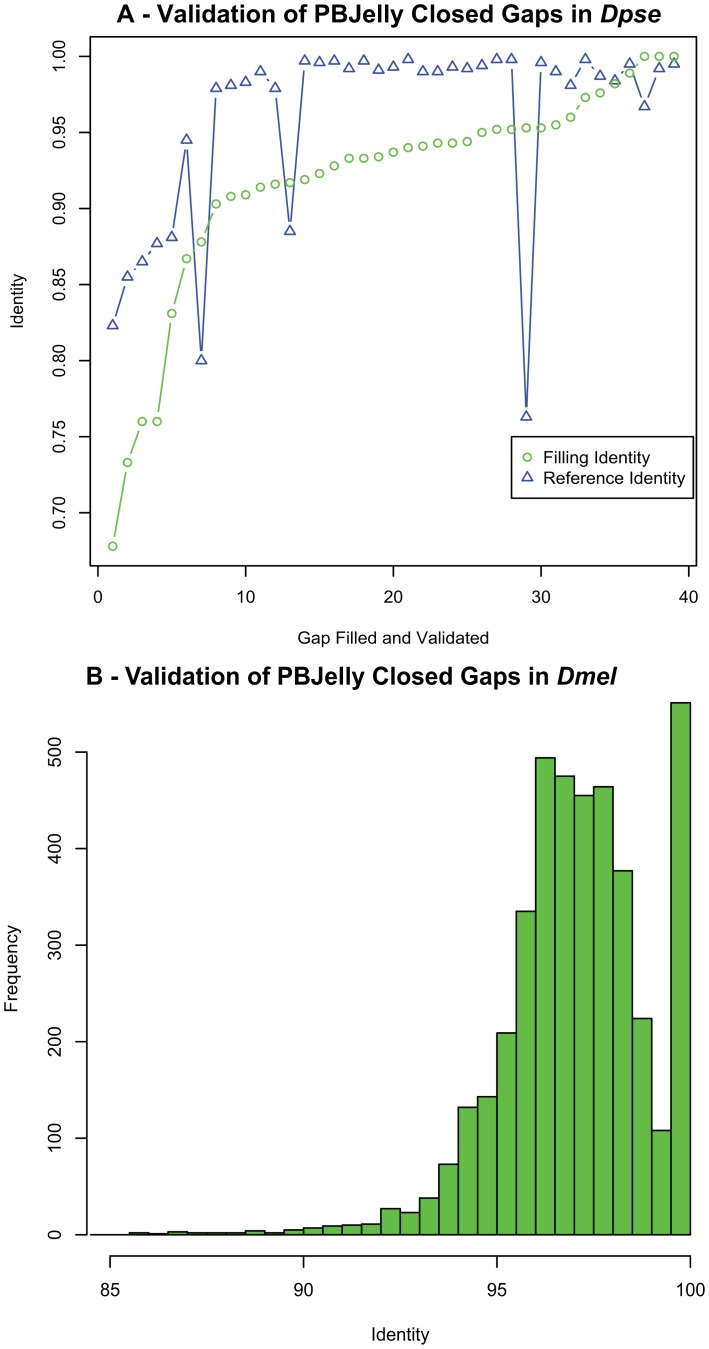
Validation of PBJelly **Results** **.** Using Sanger sequencing of Dpse we validated 7 negative gap closures (A) and 45 closed gaps (B). We also compared PBJelly's gap closing sequence with the original Dmel reference (C).

To assemble the most accurate gap consensus sequence possible from low accuracy PacBio reads we used all available gap-supporting reads. The raw percent similarity between the PacBio reads and the *Dpse* reference is an average of 81%. To assess the accuracy of our approach, we generated Sanger sequence reads for 96 gaps and compared the results to gap sequences created by PBJelly. In 45 cases reads were generated that spanned the entire original gap (Table 3). Comparing the PBJelly filling sequence with the Sanger validation sequence, the mean percent similarity is 91.7% and median percent similarity 93.7%. Since PBJelly trusts the draft genome's accuracy, we also compared the Sanger validation data with gap flanking sequence in the initial draft genome reference. This showed that the starting draft genome reference had an average of 95.9% similarity to the Sanger reads and a median 99% similarity in gap flanking regions (Figure 4a). This low quality sequence likely contributed to gap formation in the assembly. The mean percent similarity of the PBJelly gap filling sequence to the Sanger sequence was 94% in all gaps where the existing reference was better than 95% similar, but only 82% similar on average in gaps less than 95% similar to the reference.

**Table 3:** Sanger Validation Results Per Gap.

Gap Id	Flanking Contig Accuracy	Gap Filling Sequence Accuracy	Sanger #bp Over Flanking Contig	Base Pairs Placed In Gap by Sanger Read	Base Pairs Placed in Gap by PBJelly
ref0002030_110_111	82.3%	67.8%	439	111	90
ref0003044_87_88	85.5%	73.3%	539	67	72
ref0003044_71_72	86.5%	76.0%	637	90	93
ref0002030_202_203	87.7%	76.0%	467	24	24
ref0004545_3_4__	88.1%	83.1%	551	89	81
ref0003477_0_1__	94.5%	86.7%	414	233	239
ref0004673_6_7__	80.0%	87.8%	429	485	520
ref0004554_129_130	97.9%	90.3%	471	91	90
ref0004824_98_99	98.1%	90.8%	208	123	127
ref0004554_20_21	98.3%	90.9%	231	11	10
ref0004554_360_361	99.0%	91.4%	413	32	35
ref0004554_67_68	97.9%	91.6%	478	164	179
ref0000204_139_140	88.5%	91.7%	582	55	60
ref0000204_415_416	99.7%	91.9%	645	34	37
ref0003302_34_35	99.6%	92.3%	480	36	39
ref0000495_174_175	99.7%	92.8%	637	80	80
ref0003625_75_76	99.2%	93.3%	511	154	165
ref0003625_121_122	99.7%	93.3%	602	42	45
ref0003625_268_269	99.1%	93.4%	571	113	121
ref0003302_346_347	99.3%	93.7%	610	74	79
ref0000495_153_154	99.8%	94.0%	407	190	201
ref0003625_397_398	99.0%	94.1%	516	192	200
ref0003625_175_176	99.0%	94.3%	510	100	106
ref0003625_408_409	99.3%	94.3%	554	50	53
ref0003625_5_6__	99.2%	94.4%	529	204	216
ref0004554_292_293	99.4%	95.0%	506	113	119
ref0003625_91_92	99.8%	95.2%	502	99	104
ref0004554_288_289	99.6%	95.3%	540	204	210
ref0004554_300_301	99.8%	95.2%	543	118	124
ref0003302_398_399	76.3%	95.3%	277	121	127
ref0003302_675_676	99.0%	95.5%	488	63	66
ref0003441_8_9__	98.1%	96.0%	580	24	25
ref0004554_180_181	99.8%	97.3%	425	107	110
ref0000641_380_381	98.7%	97.6%	527	164	168
ref0003625_84_85	98.4%	98.2%	497	55	56
ref0002030_105_106	99.5%	98.9%	564	94	94
ref0003302_100_101	99.5%	100.0%	561	31	31
ref0002030_529_530	99.2%	100.0%	510	11	11
ref0004709_25_26	96.7%	100.0%	244	6	6
ref0003625_282_283*	98.7%	100.0%	539	−12	−12
ref0000204_291_292*	99.8%	100.0%	589	−25	−25
ref0004554_137_138*	96.7%	100.0%	426	−1	−1
ref0003044_23_24*	88.1%	100.0%	370	−43	−43
ref0002030_252_253*	99.8%	100.0%	520	−23	−23
ref0004554_118_119*	98.0%	85.7%	457	−42	−36

Negative gaps are marked with an asterisk.

Six negative gaps were Sanger validated. Negative gaps are adjacent sequence contigs that should overlap based upon estimated mate-pair distance, but do not overlap based upon the contig sequence. When comparing the number of bases trimmed from the neighboring contig sequences in PBJelly with the Sanger sequence, in all but one case PBJelly trimmed the correct bases from the neighboring contigs .

Additionally, analysis of PBJelly gap-filling sequence with the simulated *Dmel* data shows the current optimal consensus quality. Here, PBJelly-generated filling sequences had an average of 97% identity with the original reference (Figure 4b).

### Performance of PBJelly

The mapping, support, and assembly steps in PBJelly's workflow are embarrassingly parallel problems. That is to say, mapping and support of any given read, as well as assembling a gap, is an independent process for all reads or gaps respectively. Therefore, the total time PBJelly takes to upgrade an assembly is proportional to the amount of resources available to a user and the number of partitions (or jobs) one can create. The typical rate at which BLASR maps reads given 8 processors per jobs is around 300 reads/second. The support processes is single threaded and handles approximately 12500 reads/second. The slowest step is assembly, where given 4 processors for a job it takes roughly 117 seconds to assemble 18× read-coverage per gap. This means to assemble 6000 gaps sequentially (i.e. without splitting into multiple jobs) it would take just over 8 days. But, splitting the task across 10 jobs reduces the assembly time to less than a day.

### Availability

All of the code described here is available via Sourceforge at https://sourceforge.net/projects/pb-jelly/. The updated *Drosophila pseduoobscura* (Dpse_3.0) assembly has been deposited at genbank with accession number AADE00000000 and BioProject ID PRJNA10626.

## Discussion

We have presented a new tool for upgrading draft genome assemblies based on newly available long read sequence. This complements our tools Atlas-GapFill (https://www.hgsc.bcm.edu/content/bcm-hgsc-software) and Atlas-Link (https://www.hgsc.bcm.edu/content/bcm-hgsc-software), which make use of high coverage, paired end Illumina data of different insert sizes to perform a similar task. This method is widely applicable to many important draft genome references currently being used by thousands of research groups. It also allows annotations to be transferred from existing draft genome assemblies, thus greatly minimizing the additional manual curation efforts associated with new assembly versions. We expect sequences upgraded by this method will enable annotation improvements in gap-associated regions.

Currently, using only PacBio long-reads for *de novo* assembly on large genomes has not been shown to be practical due to the high (approximately 15%) error rate. Other groups have proposed correcting these reads by alignment to high quality read data [21] to enable *de novo* assembly. While these alignments are hampered in repeat regions, the approach will likely be useful in assemblies with polymorphic input data; Both of which are major causes of gaps in *de novo* genome assemblies. However, within genome contigs assembled using only current assembly tools and short-reads, base accuracy is usually high, such that PacBio reads in these overlapping regions do not contribute to sequence accuracy.

In contrast to these approaches, we align the unassembled PacBio long-reads to an existing assembly of short reads or a previous draft reference genome. Using assembled sequences has the advantage of much longer alignment lengths and thus more accurate alignments. The exceptional read length can extend into and through gaps in the original assembly, the regions most in need of attention. Both approaches are compromises to deal with the low quality of the PacBio reads in order to take advantage of their length. In the future, we hope high quality long-reads will obviate the need for either approach.

We were unable to close all of the gaps in our draft genomes. For the *Mund* assembly this was primarily due to the lower sequence coverage leaving many gaps without addressing reads. In general however we had sufficient coverage to address the vast majority of gaps, and in fact addressed 99% and 97% of gaps for *Dpse* and *Caty* respectively. However, there are multiple reasons why we might not close every gap. For the *Dpse* data the maximum gap length we expect to close is ∼2 kb with a single PBJelly iteration given an average read length of 1,228 bp and accounting for ∼200 bp to identify an alignment and an overlap of ∼150 bp between sequences extending contigs neighboring a gap. Of the 5,330 gaps below this maximum, only 4,006 (75%) of these gaps were closed. For the remaining 1,324 gaps PBJelly was able to improve 254 (19%) of the gaps, 56 gaps (4%) were unaddressed by reads, and PBJelly failed to create an assembly in 79 (6%) of these expected-to-close gaps. The last 935 (70.6%) gaps we attempted to close were flagged as being ‘overfilled’.

**Figure 5:**
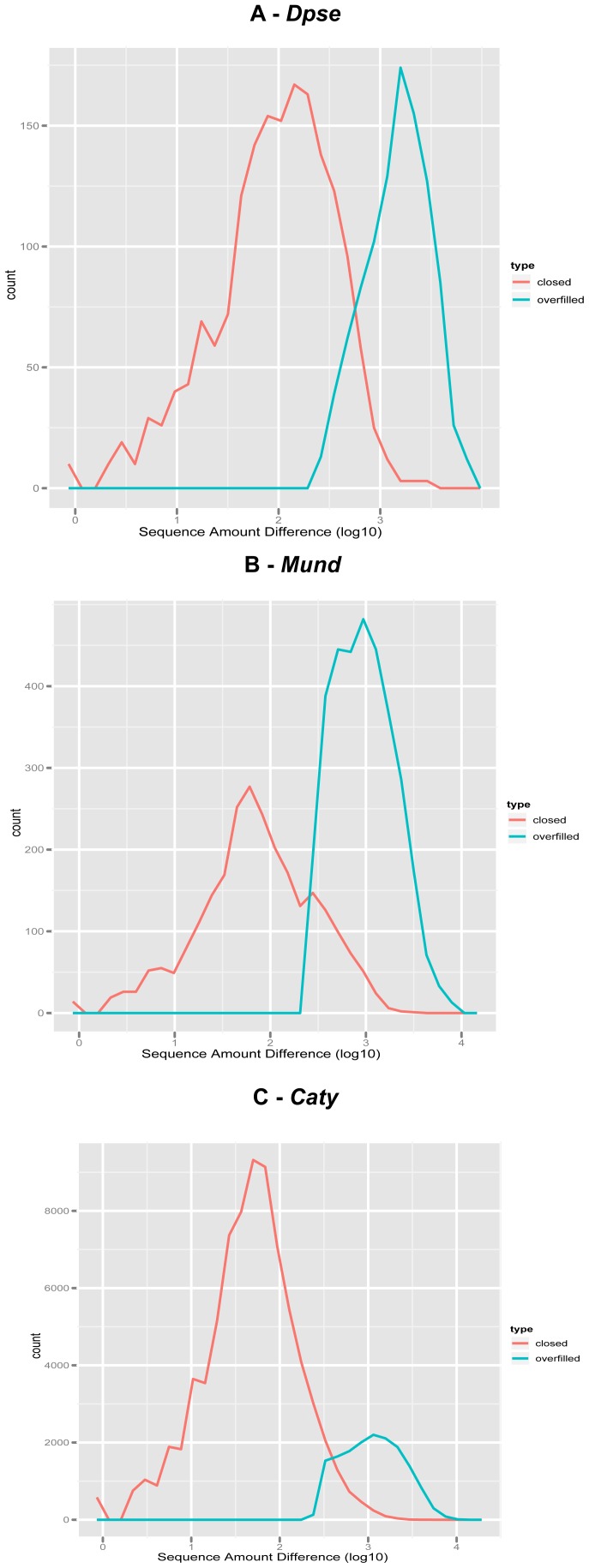
Distribution of amount of sequence placed in closed gaps compared to overfilled gaps. Frequency plots of the absolute value of sequence placed into gaps subtracted from the predicted gap size in closed gaps versus overfilled gaps in (A) Dpse (B) Mund (C) Caty. Data for Dmel is not shown because synthetically inserted gaps' predicted gap sizes matched the amount of sequence that should have been placed into the gaps.

Overfilled gaps occur when the sequences that extend either contig neighboring a gap do not have identifiable overlap, but the sum of the new sequence lengths reaching into the gap is greater than the predicted gap size. Figure 5 shows the distribution of the number of bases placed in a gap region in comparison to the predicted gap size for closed and overfilled gaps. To prevent gaps with under-estimated predicted gap sizes from being flagged as overfilled, we designed a threshold for number of bases placed into a gap before being flagged. This threshold is calculated by building a distribution from the predicted gap-size subtracted from amount of sequence placed into closed gaps, and setting our threshold at the distribution's mean plus one standard-deviation.

*Dpse* had a total of 1007 overfilled gaps. 444 (44%) were due to scaffolding errors in which the region was originally labeled as a negative gap, meaning the scaffold was incorrectly joined and should have been split. However, the remaining 563 (56%) overfilled gaps are unexplained and are suspected to be problems with the existing assembly such as inversions, under-predicted gap sizes, and pure assembly mistakes. To reduce the prevalence of this problem, increased scaffolding accuracy will need to be achieved by using more accurate scaffolding software and a higher quality/quantity of mate pair sequence information. The *Mund* and *Caty* drafts were assembled with more recently developed software than *Dpse* and consequently have better scaffolding and fewer negative gaps. This is also measured in the proportion of addressed gaps that were overfilled. The *Mund* draft had 7% overfilled gaps and the *Caty* draft had only 8.8%. In comparison, the PBJelly flagged 16.7% of all *Dpse* gaps as overfilled.

Looking forward, even longer, higher quality reads are promised by both Pacific Biosystems (Carlsbad CA) and Oxford Nanopore (Oxford UK). PBJelly requires only fasta format sequences for input, and will be able to immediately utilize such data, making this approach more powerful. Moreover, it is possible for even longer, higher quality reads to improve *de novo* assemblies to such an extent as to make this upgrade technique obsolete in favor of replacement assemblies. Until that happens, PBJelly with current long read data provides real improvements to highly used reference sequences with little additional annotation work for the model-organism communities that rely on them.

### Future Directions

We plan a number of improvements for future versions of PBJelly. (1) The assembly techniques can be improved by incorporating the sequence similarity to flanking contigs to adjust the predicted error rate. This will help the make-consensus step in AMOS. PBJelly assumes a 15% error rate for each iteration of assembly, although assembled contig error rates are lower than the error rates for the initial reads. (2) Discovering and implementing better assessment metrics for deciding the fitness of contigs created from the set of supporting reads for a gap. (3) Another goal is to extend PBJelly's capability from filling captured gaps to improving scaffolding by uniting unanchored contigs into scaffolds. (4) Currently, contig-end trimming around negative gaps relies on continuous alignments to the gap adjacent sequences, but alignments from the fast aligner BLASR often contain mismatches within the negative gap. Using local realignment would reduce mismatches and improve the repairs of these negative gaps. (5) Finally, improvements are planned to make use of much longer sequence reads (∼100 kb reads are possible from nanopore sequencing technologies) such as incorporating other alignment programs for the mapping stage.

In addition to continuing support and development of PBJelly, we will explore the characteristics of overfilled gaps. While overfilled gaps are the result of errors in the original assembly process, we are interested in understanding why these problems exist. Furthermore, how can we use long-read technologies to correct these errors and produce even higher-quality genomes?

## Materials and Methods

### Ethics Statement

This study did not require any procedures to be performed on live animals. All DNA samples used were obtained from existing stocks held as frozen aliquots from prior studies. Consequently, no live animals were used and no animal welfare issues are relevant.

### *Dpse* DNA isolation

DNA was isolated for the original genome sequencing of *Dpse (14)* ∼3 mg of this DNA was stored in TE at −20°C for approximately 8 years before library construction for PacBio sequencing. This avoided issues of polymorphism between different *Dpse* strains, but more recent DNA isolations have generated longer PacBio reads, we presume due to the absence of single stranded breaks generated during storage.

### PacBio Library construction and DNA sequencing

Genomic DNA was sheared to 8 kb using an ultrasonicator (Covaris Inc, Woburn, MA) and was converted into the proprietary SMRTbell™ library format using RS DNA Template Preparation Kit (Pacific Biosciences, Melon Park, CA). Briefly, sheared DNA was end repaired, and hairpin adapters were ligated using T4 DNA ligase. Incompletely formed SMRTbell templates were degraded with a combination of Exonuclease III and Exonuclease VII. The resulting DNA templates were purified using SPRI magnetic beads (AMPure, Agencourt Bioscience, Beverly, MA) and annealed to a two-fold molar excess of a sequencing primer that specifically bound to the single-stranded loop region of the hairpin adapters.

SMRTbell templates were subjected to standard SMRT sequencing using an engineered phi29 DNA polymerase on the PacBio RS system according to manufacturer's protocol. The PacBio RS system continuously monitors zero-mode waveguides (ZMWs) in sets of 75000 at a time. Within each ZMW a single DNA polymerase molecule is attached to the bottom surface such that it permanently resides within the detection volume where it can be watched as it performs sequencing by synthesis. Within each chamber, Phospholinked nucleotides, each type labeled with a different colored fluorophore, are then introduced into the reaction solution at high concentrations that promote enzyme speed, accuracy, and processivity. Pulse calling, utilized a threshold algorithm on the dye weighted intensities of fluorescence emissions, and read alignments, achieved using a Smith-Waterman algorithm. Reads were filtered after alignment to remove low quality sequences derived from doubly-loaded ZMWs.
